# Design Approach for Reducing Cross-Axis Sensitivity in a Single-Drive Multi-Axis MEMS Gyroscope

**DOI:** 10.3390/mi12080902

**Published:** 2021-07-29

**Authors:** Hussamud Din, Faisal Iqbal, Byeungleul Lee

**Affiliations:** School of Mechatronics Engineering, Korea University of Technology and Education, Cheonan 31253, Korea; hussam@koreatech.ac.kr (H.D.); faisal@koreatech.ac.kr (F.I.)

**Keywords:** microelectromechanical systems (MEMS), single-drive, multi-axis, gyroscope, cross-axis sensitivity, finite element analysis (FEA) model, COMSOL

## Abstract

In this paper, a new design technique is presented to estimate and reduce the cross-axis sensitivity (CAS) in a single-drive multi-axis microelectromechanical systems (MEMS) gyroscope. A simplified single-drive multi-axis MEMS gyroscope, based on a mode-split approach, was analyzed for cross-axis sensitivity using COMSOL Multiphysics. A design technique named the “ratio-matching method” of drive displacement amplitudes and sense frequency differences ratios was proposed to reduce the cross-axis sensitivity. Initially, the cross-axis sensitivities in the designed gyroscope for x and y-axis were calculated to be 0.482% and 0.120%, respectively, having an average CAS of 0.301%. Using the proposed ratio-matching method and design technique, the individual cross-axis sensitivities in the designed gyroscope for x and y-axis were reduced to 0.018% and 0.073%, respectively. While the average CAS was reduced to 0.045%, showing a reduction rate of 85.1%. Moreover, the proposed ratio-matching method for cross-axis sensitivity reduction was successfully validated through simulations by varying the coupling spring position and sense frequency difference variation analyses. Furthermore, the proposed methodology was verified experimentally using fabricated single-drive multi-axis gyroscope.

## 1. Introduction

Microelectromechanical systems (MEMS) gyroscopes have recently received growing attention due to their compact size, low price, and easy integration with mainstream complementary metal-oxide-semiconductor (CMOS) technology. The market trend of MEMS devices, specifically for gyroscopes, has almost doubled in the last five years, and will be tripled in the next two years, while the market value in consumer electronics has increased by 11.2% since 2013. This high market demand for MEMS gyroscopes is mainly due to its promising application potential in the military, aviation, consumer electronics, navigation systems, medical, automobile, and robotics fields [1,2,3,4,5,6,7,8,9,10,11].

In the last few decades, meticulous research has been performed for design and performance improvement to expand MEMS gyroscope applications further. Some of the MEMS industry’s challenges are design miniaturization and structure simplicity, including both the MEMS mechanical part and circuit integration. Besides this, the gyroscope’s sound performance is vital, particularly in terms of sensitivity and cross-axis sensitivity, to meet today’s challenging industry specifications [12,13]. Manufacturing uncertainties always exist due to the limitations of the design principle and the machining accuracy and are relatively more numerous in MEMS devices than macro-scale devices. Such uncertainties affect the performance of the MEMS devices [14,15]. Cross-axis sensitivity (CAS) is a significant issue in a single-drive multi-axis MEMS gyroscope due to the performance specifications. A variety of research has been performed to investigate the primary sources responsible for producing cross-axis sensitivity and estimating and reducing them. CAS in MEMS gyroscope occurs either due to frontend errors, related to mechanical structure and design issues, or backend errors, related to assembly and testing setup [12,15,16,17,18].

Various multi-axis MEMS gyroscopes has been presented in the literature. A single-proof mass dual-axis gyroscope design was presented, with x and y-axis cross-axis sensitivities of 25.2% and 20.1%, respectively, resulting due to quadrature error [19]. Experimental evaluation of tri-axis MEMS gyroscope was presented, and cross-axis sensitivities for x/y-axis and z-axis were measured to be 22%, 9%, and 1.84%, respectively, resulting from the mechanical coupling of the masses [20]. A dual-axis mode matched micro-machined rate gyroscope was presented with experimentally measured cross-axis sensitivities ranging from 3% to 16%, and claimed that a part of this cross-axis sensitivity is due to the slight frequency difference and the primary source is the mechanical cross-coupling [21]. A design of a 3-axis single chip MEMS gyroscope was presented and analyzed through simulations using Ansys software, whereas cross-axis sensitivity was computed below 2.82% [22]. A 3-axis MEMS gyroscope was presented with a catch-and-release structure with four coupled drive masses with confirmed cross-axis sensitivity of less than 2% [23]. An experimental 3D test-setup, presented for evaluating multi-axis MEMS gyroscope and cross-axis sensitivity for x, y, and z gyroscopes, was measured to be 1.75%, 1.67%, and 0.37%, respectively, and 3-D fixture design angles were considered the main error source for cross-axis sensitivity [24]. A MEMS surface micro-machined gyroscope with 0.70% cross-axis sensitivity was presented and claimed that this small value of CAS is due to the large frequency split between operational and out-of-plane frequencies [25]. A compact 3-axis MEMS gyroscope with decoupled structure was presented, and cross-axis sensitivity of less than 0.30% was experimentally measured for all three axes [26].

This work’s main objective is to develop a design technique to estimate and reduce the cross-axis sensitivity in a single-drive multi-axis MEMS gyroscope using COMSOL Multiphysics. For a single-drive multi-axis MEMS gyroscope having a complex mechanical-structure, it was difficult to estimate and reduce the CAS using MATLAB or analytical approaches. However, COMSOL Multiphysics offers a more straightforward approach, relatively more accurate and informative than other approaches for CAS analysis. In this work, a previously designed simplified single-drive multi-axis MEMS gyroscope utilizing a mode-split approach [27] was analyzed for CAS using the established finite element analysis (FEA) based methodology [28]. A two-point measurement method for cross-axis displacement in the designed gyroscope was introduced. A design technique named the “ratio-matching method” was proposed and implemented to reduce the CAS in a single-drive multi-axis MEMS gyroscope. In the proposed design technique, the ratio of drive displacement amplitudes vs. the sense frequency differences was normalized to reduce the CAS. The average cross-axis sensitivity in the designed gyroscope was reduced by 85.1%, and x-axis and y-axis CAS were individually computed as 0.018% and 0.073%, respectively. The proposed method for CAS reduction was successfully validated through simulations and experimentally.

This paper is structured as follows. In Section 2, the mechanical structure of the designed single-drive multi-axis MEMS gyroscope is presented. In Section 3, cross-axis sensitivity modeling in the designed gyroscope is discussed in detail. Section 4 presents the design approach. In Section 5, the simulation and experimental results are discussed, followed by the conclusion and future work in Section 6.

## 2. Mechanical Structure of the Designed Gyroscope

**Figure 1:**
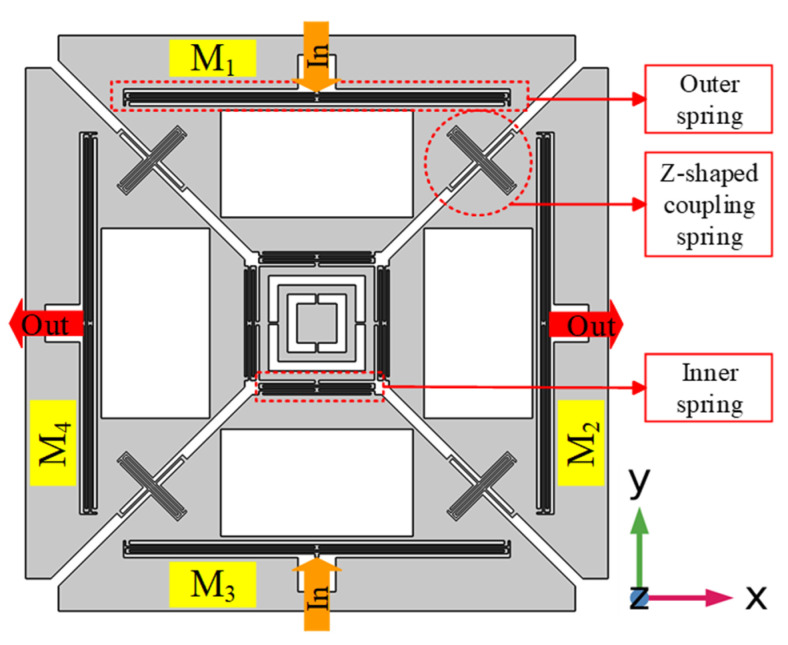
2D Layout of the Designed Microelectromechanical systems (MEMS) Gyroscope.

**Figure 2:**
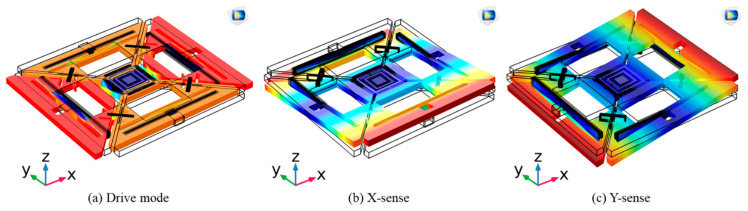
Resonant Modes of the Designed MEMS Gyroscope.

The single-drive multi-axis MEMS gyroscope structure was previously designed to consist of four masses named $M_{1}$, $M_{2}$, $M_{3}$, and $M_{4}$, coupled by a Z-shaped coupling spring as shown in Figure 1 [27], whereas the resonant modes are shown in Figure 2. The inner double-folded springs along with the coupling springs are responsible for the in-plane drive motion. During the drive motion of the designed gyroscope, each axis has two masses, moving opposite to each other, (i.e., $M_{1}$ and $M_{3}$ move inward while $M_{2}$, and M_4_ move outward). Drive-scheme of the designed gyroscope has the ability to reduce the slide-film damping and keep the unwanted resonant modes at higher frequencies. The Z-shaped coupling spring can limit the drive displacement amplitude in one of the two drive axes; i.e., x-masses drive displacement *x*_*dx*_ and y-masses drive displacement *x*_*dy*_ are not equal [29,30]. The outer double-folded springs are responsible for sense-motion, however $M_{1}$ and $M_{3}$ correspond to angular rate in x-axis they were named as x-masses, whereas $M_{2}$ and $M_{4}$ were named as y-masses, which corresponds to the angular rate in y-axis as shown in Figure 2b,c, respectively.

## 3. Cross-Axis Sensitivity Modeling

### 3.1. Gyroscope’s Cross-Axis Sensitivity

Cross-axis sensitivity for a gyroscope is defined as the sensor output on a sense-axis due to the applied input angular rate in the orthogonal axis expressed in percentage. Mathematically, cross-axis sensitivity for a three-axis gyroscope can be described by Equation (1) [24,31].

(1) $$S_{i,j/k_{Cross}}=\left(\frac{\sqrt{(S_{ij}^{2}+S_{ik}^{2}})}{S_{ii}}\right)\times 100\;\%$$

The first subscript represents the primary axis in which the angular rate was measured, and the second subscript represents the axis in which the external angular rate was applied. Considering only x-axis and y-axis, Equation (1) for cross-axis sensitivity reduced to Equation (2)

(2) $$S_{i,j_{Cross}}=\left(\frac{S_{ij}}{S_{ii}}\right)\times 100\;\%$$

### 3.2. Cross-Axis Sensitivity Modeling in the Designed Gyroscope

For an ideal design of a MEMS gyroscope, the theoretical cross-axis sensitivity should be zero, which is not a case in practical devices. However, CAS can be minimized to a value nearing to zero by using a proper symmetric and balanced design. In simulations, CAS in multi-axis MEMS gyroscope cannot be completely eliminated due to its complex mechanical structure with several critical elastic elements, asymmetries, and lack of computational resources; however, it can be minimized to a value near zero [32].

**Figure 3:**
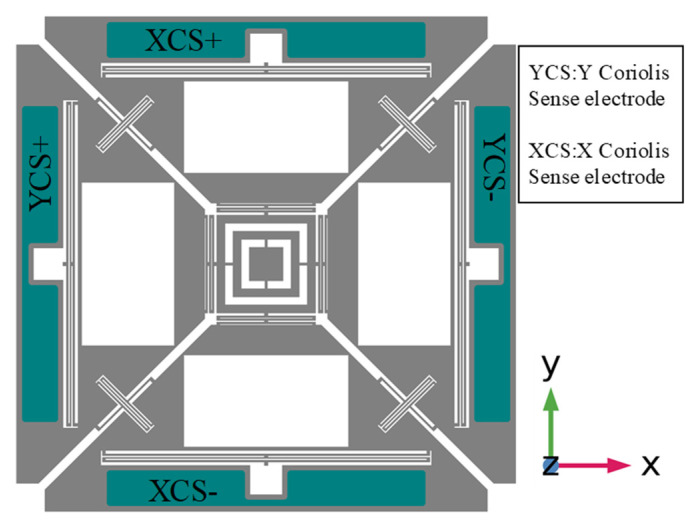
Bottom Electrode Layers of the Designed MEMS Gyroscope.

**Figure 4:**
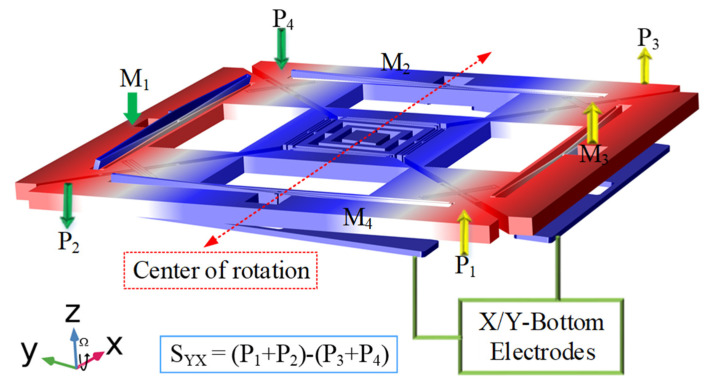
Cross-axis Displacement Measurement of the Designed MEMS Gyroscope.

In the designed multi-axis gyroscope, x and y-masses were coupled by a coupling spring. Therefore, the orthogonally coupled masses also move by the Coriolis force and exhibited out-of-plane motion due to primary sense masses motion. In the designed gyroscope, x and y Coriolis sense (XCS/YCS) electrodes were placed under their respective masses and are aligned at the center, as shown in Figure 3. Out-of-plane sense motion of the proposed design resembles a seesaw motion; i.e., when the input angular rate is applied in x-axis, then x-masses exhibit out-of-plane motion as main Coriolis motion, (i.e., $M_{1}$ moved downward and $M_{3}$ moved upward). The orthogonally coupled y-masses also experienced out-of-plane motion, and each mass (i.e., $M_{2}$ and $M_{4}$) individually exhibited a seesaw motion due to cross-coupling phenomena. It can be described by considering the points as $P_{3}$ of $M_{2}$ moved upward and P_4_ moved downward, and similarly P_1_ of $M_{4}$ moved upward while P_2_ of M_4_ moved downward, as shown in Figure 4.

The total cross-axis displacement of an axis can be measured by a 2-point measurement method, as each cross-coupled sense mass exhibited seesaw motion. Displacements of the two points (i.e., $P_{1}$ and $P_{2}$ of each mass $M_{4}$) should be measured and added together, while P_3_ and $P_{4}$ of the other mass M_2_ should be added, and then the differential of these summations (i.e., “( $\left(P_{1}+P_{2})-(P_{3}+P_{4}\right)$ )”) resulted in the total cross-axis displacement of y-axis due to x-axis as shown in Figure 4.

**Figure 5:**
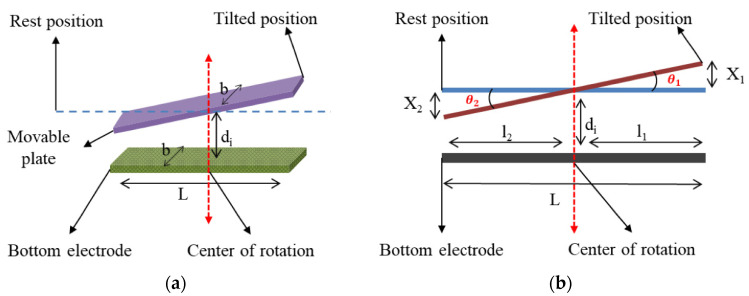
Inclined Plate Capacitor in Tilted Position. (**a**) Schematic View of Inclined Capacitor; (**b**) Free Body Diagram of Inclined Capacitor.

The phenomena of cross-axis motion in the designed gyroscope can be better understood by comparing it with an inclined plate capacitor as the bottom electrodes were aligned at center with the sense electrodes, as shown in Figure 5a,b. Equation (3) describes the mathematical expression for the capacitance of an inclined plate capacitor [33].

(3) $$C_{inc}=\frac{\varepsilon_{o}b}{d_{i}}\left[\frac{1}{2d_{i}}\left(l_{2}^{2}\theta_{2}-l_{1}^{2}\theta_{1}\right)+\left(l_{1}+l_{2}\right)\right]$$

where $C_{inc}$ is the capacitance of the inclined plate capacitor, $\varepsilon_{o}$ is the free space permittivity, *d*_*i*_ is the initial electrode gap, *b* is the electrode width, $l_{1}$ and $l_{2}$ are the side lengths, half of the total length “L” of the electrode, respectively, and $\theta_{1}$ and $\theta_{2}$ are the corresponding angles related to $X_{1}{\;\mathrm{and}\;X}_{2}$ displacements, respectively, as shown in Figure 5b.

The total capacitance of parallel plate and inclined plate capacitors are the same when $\theta_{1}=\theta_{2}$, or $X_{1}=X_{2}$. However, a change in capacitance occurs if the inclined plate capacitor’s angles or displacements are different, similar to the cross-axis motion in the designed single-drive multi-axis MEMS gyroscope. Change in capacitance is given by Equation (4).

(4) $$\Delta_{C_{inc}}=C_{inc_{Rest}}-C_{inc_{Final}}$$

In this equation, $\Delta_{C}{}_{inc}$ is the change in capacitance, $C_{inc_{Rest}}$ is the rest capacitance, and $C_{inc_{Final}}$ is the final capacitance of the inclined plate capacitor.

## 4. Design Approach

**Figure 6:**
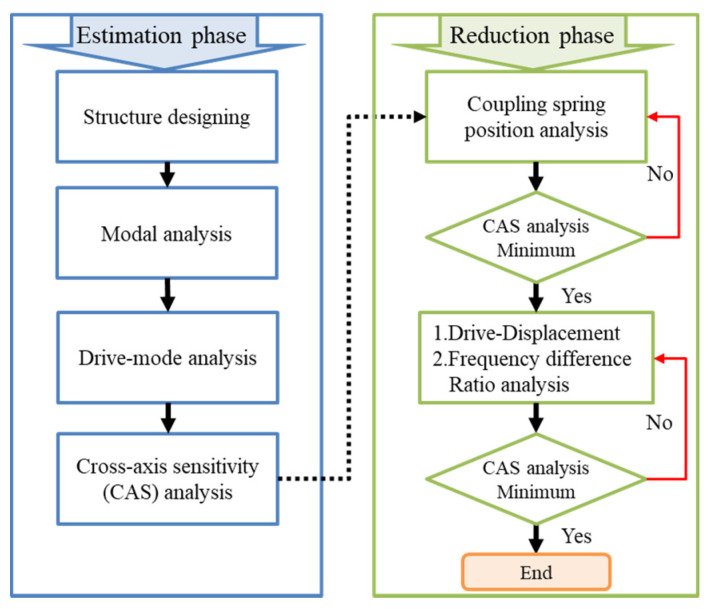
Proposed Design Approach Workflow for Cross-Axis Sensitivity Analysis for the Designed Gyroscope.

The proposed design approach for estimating and reducing the cross-axis sensitivity of a single-drive multi-axis MEMS gyroscope was based on a previous published work and FEA methodology using COMSOL Multiphysics [28]. This design approach consisted of two phases, (i) estimation phase and (ii) reduction phase of cross-axis sensitivity in the designed gyroscope, as shown in the workflow Figure 6. The CAS estimation phase consisted of several FEA analyses, including structure designing, modal analysis, drive, and sense mode analyses, which is explained in detail in our previous work [28]. In sense mode analysis, cross-axis sensitivity was estimated using two-point method. Reduction phase of CAS consisted of the coupling spring position analysis, and drive displacement amplitudes ratio and sense frequency difference ratio analysis. Initially, an optimum position for coupling spring having minimum CAS was achieved through parametric analysis of coupling spring position, then further analysis for CAS reduction was performed using the proposed technique of “ratio-matching method” of drive displacement amplitudes and sense frequency differences ratios.

### 4.1. Numerical Analysis of Cross-Axis Sensitivity in the Designed Gyroscope

The designed gyroscope was simulated using solid mechanics module of COMOSOL Multiphysics. All the analyses were performed using the “user-controlled mesh” with extremely fine size having maximum element size 18.5 μm and minimum element size 0.185 μm. Modal analysis of the designed gyroscope was performed to determine the resonant frequencies and their mode shapes listed in Table 1. Drive mode analysis was performed depicting 3.52 μm and 4.06 μm drive displacement amplitudes for x and y-masses, respectively, as a result of applied harmonic driving force *F_d_* = 0.36 μN, using the developed FEA methodology. The drive displacement amplitudes difference is due to the Z-shaped coupling spring.

To estimate the cross-axis sensitivity in the designed single-drive multi-axis MEMS gyroscope, Coriolis-mode analysis was performed. The designed single-drive multi-axis MEMS gyroscope was driven at its driving resonant frequency of 25,682 Hz, and then angular input rate of 2000 degrees per second (dps) was applied to the structure by adding a rotatory frame to the study. A drive frequency sweep of 25,675–25,689 Hz having a resolution of 0.1 Hz was added to the study, and Coriolis-response plots of x-sense and y-sense were computed according to the applied input angular rate in their respective axes, showing sense displacements as well as cross-axis displacements.

**Figure 7:**
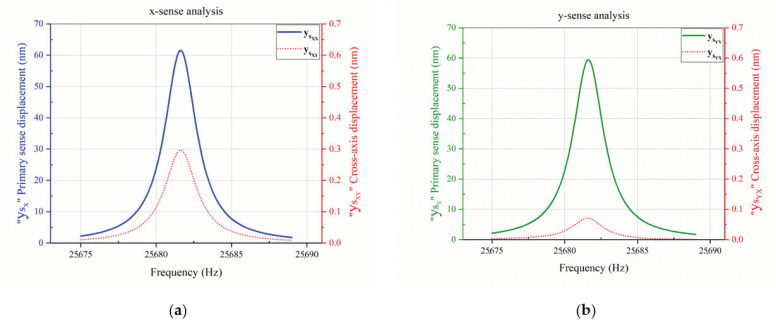
Cross-axis Sensitivity Analysis of the Designed MEMS Gyroscope. (**a**) x-sense analysis; (**b**) y-sense analysis.

Figure 7a shows the Coriolis response plot of x-sense, depicting the total differential sense displacement on the primary axis and the cross-axis displacement calculated by the two-point measurement method, on the secondary axis of the plot. For x-sense displacement, input angular rate was applied in x-axis, while for cross-axis displacement, input angular rate was applied in y-axis. Similarly, Figure 7b shows the Coriolis response plot of y-sense, depicting the total differential sense displacement on the primary axis and cross-axis displacement calculated using the two-point measurement method on the secondary axis of the plot. For y-sense displacement, input angular rate was applied in y-axis, while for cross-axis displacement, input angular rate was applied in x-axis. Using Equation (2), x and y-axes cross-axis sensitivities were calculated to be 0.482% and 0.120%, respectively, having an average CAS of 0.301%, as listed in Table 2. To reduce the existing cross-axis sensitivity, several analyses were performed using the proposed methodology, as discussed in the next sections.

### 4.2. Effect of Coupling Spring Position on the CAS in the Designed Gyroscope

**Figure 8:**
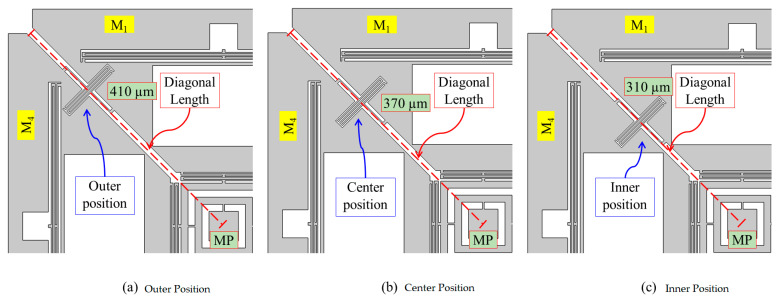
Coupling Spring Position of the Designed MEMS Gyroscope.

**Figure 9:**
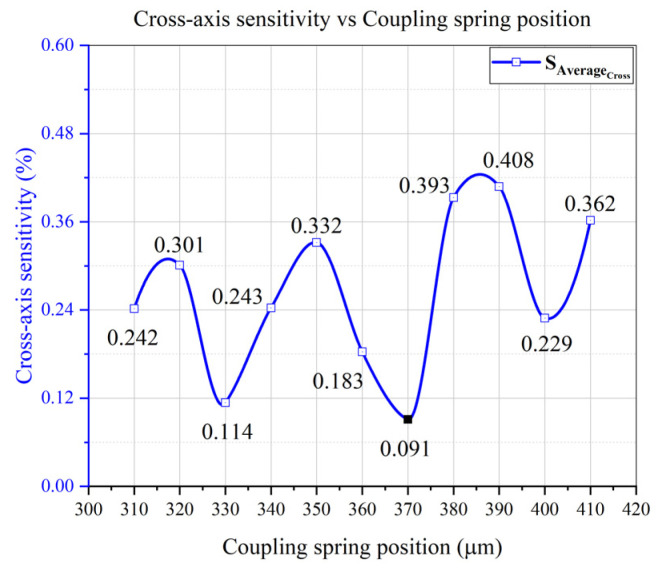
Cross-Axis Sensitivity vs. Coupling Spring Position Analysis of the Designed MEMS Gyroscope.

In MEMS devices, cross-axis sensitivity is caused by asymmetrical structural design. Therefore, symmetrical structure is important for low CAS, and can be achieved by adjusting and aligning the center of masses of the coupling spring and proof mass [34]. Initially, the effect coupling spring on cross-axis sensitivity was analyzed, as it couples the x- and y-sense masses. In the designed gyroscope, a Z-shaped coupling spring was utilized and placed at 45° between the x- and y-masses, according to the structure of the sense masses. The coupling spring was placed at different points from innermost to outermost possible location after every 10 μm, between x- and y-sense masses. Position of the coupling spring can be measured through the diagonal length from the middle point (MP) of the design, as shown in Figure 8. Each structure of the designed gyroscope having coupling spring at different position was simulated including modal analysis, drive and sense mode analysis, and cross-axis sensitivity was computed. The CAS analysis results of all the structures were summarized, and the average cross-axis sensitivity was plotted as shown in Figure 9. The results depicted no specific trend for CAS reduction; however, a minimum cross-axis sensitivity of 0.091% was achieved at the center position for the coupling spring placement. The fluctuation in Figure 9 is due to the structural asymmetries in the design. However, minimum CAS at the center position reveals that symmetrical structural design has been achieved.

The coupling spring position variation analysis showed that the cross-axis sensitivity of the designed gyroscope depended on the coupling spring position. The center position of the x and y-coupled masses was considered as an optimum position for coupling spring placement, based on the minimum average cross-axis sensitivity. Further analysis to investigate and reduce the cross-axis sensitivity in the designed gyroscope was performed, keeping the coupling spring at the center position as discussed in the next sections.

### 4.3. Drive Displacement Amplitudes vs. Sense Frequency Differences Analysis

On account of further investigation of cross-axis sensitivity of MEMS gyroscope, relationship of gyroscope main sensitivity, drive displacement, and sense frequency difference was analyzed. It can be seen that main sensitivity “$y_{s}$” of the MEMS gyroscope directly related to the drive displacement amplitude “$x_{d}$”, and inversely to the sense frequency difference “Δω”, as described in Equation (5) [28,32].

(5) $$y_{s}=\frac{2\Omega \omega_{d}x_{d}}{\sqrt{{\left[\omega_{s}^{2}-\omega_{d}^{2}\right]}^{2}+{\left[\frac{\omega_{d}}{Q_{s}\omega_{s}}\right]}^{2}}}$$

where Ω is the angular input rate, $\omega_{d}$ and $\omega_{s}$ are the drive and sense frequencies, respectively, $x_{d}$ is the drive displacement amplitude, and $Q_{s}$ is the sense quality factor.

The cross-axis sensitivity term of x-axis “$S_{ij}$” has a direct relation with the primary sensitivity term of y-axis “$S_{jj}$”. Similarly, the cross-axis sensitivity term of y-axis “$S_{ji}$” has direct relation with the primary sensitivity term of x-axis “$S_{ii}$”, as discussed in Section 3.1 and described in Equation (2). This statement means that if x-axis sensitivity “$S_{ii}$” is high, then the cross-axis sensitivity term for the orthogonally coupled y-axis “$S_{ji}$”, will be high, and hence result in high cross-axis sensitivity in y-axis, “$S_{j_{Cross}}$” for a lower value of y-axis sensitivity “$S_{jj}$”. To rectify this situation, the main sensitivities of the coupled x-axis and y-axis need to be equal in order to keep the cross-axis sensitivity terms equal (i.e., $S_{ii}=S_{jj}$, then $S_{ji}=S_{ij}$).

In the mode-split design approach of a gyroscope, sense frequencies are always different and deviate from drive mode, making the main sensitivities different. According to the above discussion, this frequency difference is also responsible for cross-axis sensitivity, and is faintly discussed in the previous literature [21,25]. In the mode-split design approach, it is difficult to match the main sensitivities, as well as cross-axis sensitivities, in a straightforward manner. The only possible way is to deal with the drive displacement amplitudes, and limit them according to sense frequency differences. This statement and analysis result in a unique relation of drive displacement amplitudes and sense frequency differences to reduce cross-axis sensitivity in the form of a “ratio-matching method”. For example, the sizeable drive displacement amplitude is necessary for an axis having higher sense frequency or large sense frequency difference with the drive frequency, while the low drive displacement amplitude is required for an axis having lower sense frequency or small sense frequency difference to normalize the ratios. This methodology of drive displacement amplitudes and sense frequency differences can be better understood by the following Equation (6).

(6) $$\left\{\begin{array}{c} S_{xx}=S_{yy}\\ S_{xy}=S_{yx} \end{array};\;\frac{↑x_{d_{X}}}{↑\Delta \omega_{x}}\;and\;\frac{↓x_{d_{Y}}}{↓\Delta \omega_{y}}\right\}$$

In this equation, $S_{xx}$ and $S_{yy}$ are x and y-axis main sensitivities and $S_{xy}$ and $S_{yx}$ are the x and y-axis cross-axis sensitivity terms, whereas $x_{d_{X}}$ and $x_{d_{Y}}$ are x and y-masses drive displacements, and $\Delta \omega_{x}$ and $\Delta \omega_{y}$ are x and y-sense frequency differences, respectively.

The drive-scheme of the designed single-drive multi-axis MEMS gyroscope generating different drive displacement amplitudes for both x and y-axes, and this was achieved with the use of a unique Z-shaped coupling spring which can limit the drive displacement amplitudes in one of the two coupled axes; i.e., $x_{d_{X}}\neq x_{d_{Y}}$ [27]. For an optimized structure with low cross-axis sensitivity, it is suggested that drive displacement amplitudes ratio “$R_{x_{d}}”$, described in Equation (7), and sense frequency difference ratio “$R_{\Delta_{\omega }}”$, described in Equation (8), should be same, or their ratio should be equal or approaching to 1, as described in Equation (9).

(7) $$R_{x_{d}}=x_{d_{X}}/x_{d_{Y}}$$

(8) $$R_{\Delta_{\omega }}=\Delta \omega_{x}/\Delta \omega_{y}$$

(9) $$Ratio=\left|\frac{R_{x_{d}}}{R_{\Delta_{\omega }}}\right|\equiv 1$$

**Figure 10:**
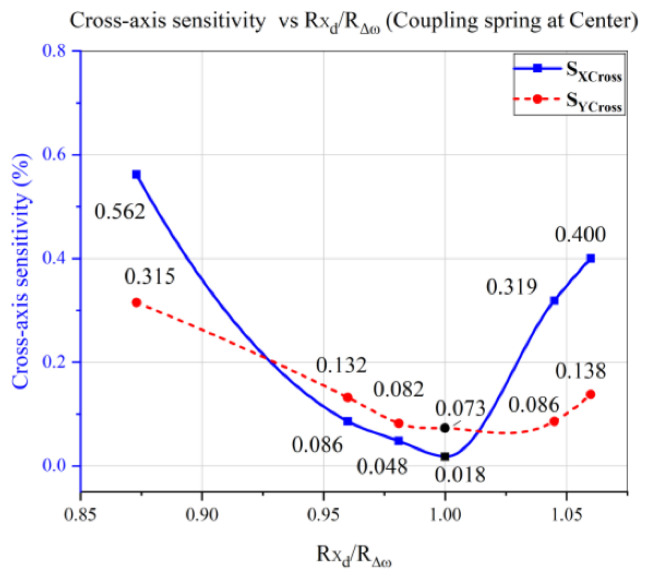
Cross-Axis Sensitivity vs. $R_{x_{d}}/R_{\Delta_{\omega }}$ Analysis of the Single-Drive Multi-Axis MEMS Gyroscope.

Several analyses were performed for computing cross-axis sensitivity using the “ratio-matching method” while the coupling spring was kept at center position. Figure 10 shows that the cross-axis sensitivity for x and y-axes was reducing whenever the ratio “$R_{x_{d}}/R_{\Delta_{\omega }}$” was approaching 1, and minimum CAS was noted to be 0.018% and 0.073%, respectively.

**Figure 11:**
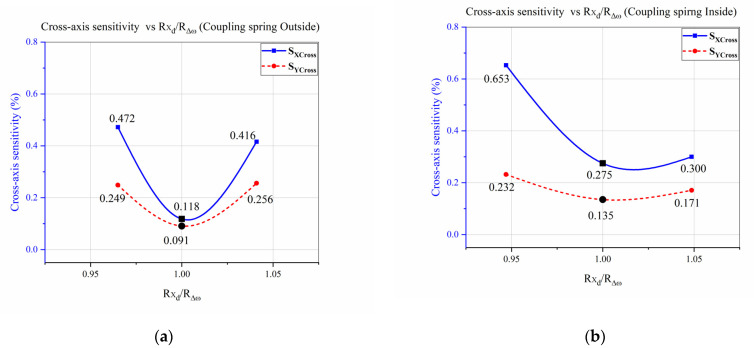
CAS vs. “$R_{x_{d}}/R_{\Delta_{\omega }}$” w.r.t. Coupling Spring Position of the Designed MEMS Gyroscope. (**a**) Coupling Spring at the Outer Side; (**b**) Coupling Spring at the Inner Side.

To verify the proposed “ratio-matching method” for CAS reduction, the same analysis was performed, keeping the coupling spring at the innermost and outermost end of the sense masses. Figure 11a,b shows cross-axis sensitivity analysis concerning coupling spring position variation and demonstrates that CAS was decreasing whenever the ratio “$R_{x_{d}}/R_{\Delta_{\omega }}$” approached 1.

It was concluded from the analyses shown in Figure 10 and Figure 11 that CAS was reducing whenever the ratio “$R_{x_{d}}/R_{\Delta_{\omega }}$” approached 1, regardless of the coupling spring position. Hence, the above analyses show that the CAS of a single-drive multi-axis MEMS gyroscope was related to the sense frequency difference, controlled by the drive displacement amplitude through the proposed “ratio-matching method”. However, for a single-drive multi-axis MEMS gyroscope with low cross-axis sensitivity, it was necessary to place the coupling spring at the center position of the sense masses, and design the drive scheme according to the sense frequency differences by utilizing a Z-shaped coupling spring.

## 5. Results and Discussion

### 5.1. Simulation Results

The designed single-drive multi-axis MEMS gyroscope was simulated for cross-axis sensitivity and initially the average CAS was measured to be 0.301%. To reduce cross-axis sensitivity, a coupling spring position was targeted. The coupling spring was placed at different positions and CAS analysis was performed, resulting in average CAS of 0.091% at the center position. These analyses reveal that the coupling spring should be placed at center of the x–y coupled masses to achieve minimum CAS. Cross-axis sensitivity was further reduced by proposed “ratio-matching method” of drive displacement amplitudes and sense frequency difference ratios. Using “ratio-matching method” the average cross-axis sensitivity was successfully reduced 0.045%, while individual x- and y-axes’ CAS was measured to be 0.018% and 0.073%, respectively. The results of “ratio-matching method” analyses reveal that normalizing the sense frequency difference with respect to driving frequency, of a mode-split single-drive multi-axis MEMS gyroscope, has significantly improved the cross-axis sensitivity. According to the literature, in practical devices, CAS in multi-axis MEMS gyroscope cannot be completely eliminated, however it can be minimized, as was successfully achieved in the proposed design.

### 5.2. Experimental Results

Furthermore, the proposed “Ratio-matching method” for cross-axis sensitivity reduction was successfully validated experimentally. The experiments were performed on the previously fabricated multi-axis MEMS gyroscope using the same experimental setup. The detailed structure design and experimental arrangement is presented in [29].

Initially, the drive amplitude ratio was calculated from the time domain signal after removing the parasitic feed-through signal [35]. The fabricated device utilizes the Z-coupling spring, which constitutes different drive displacements for the x and y-sense masses. Furthermore, the x and y-sense resonant frequencies were electrically tuned for ratio matching.

**Figure 12:**
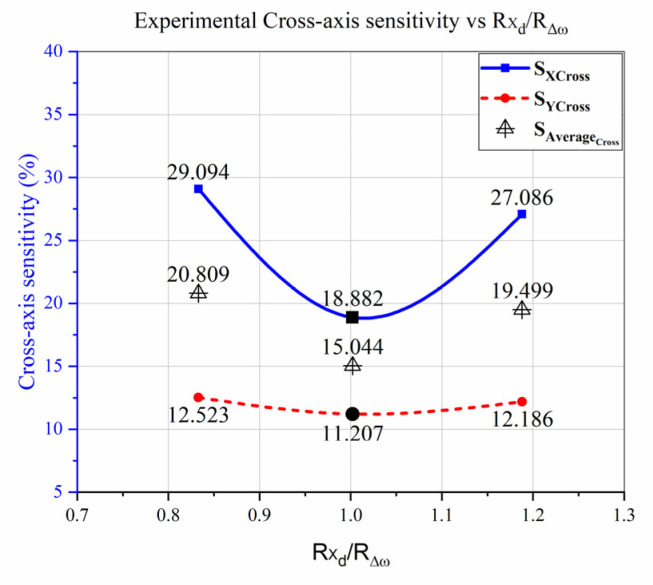
Experimental CAS vs. “$R_{x_{d}}/R_{\Delta_{\omega }}$” of the Previous Fabricated Designed MEMS Gyroscope.

Figure 12 reveals the experimental results, depicting the cross-axis sensitivity of the fabricated device, which is comparatively high. The experimentally tested device was previously fabricated with a different design, although the previous design did not follow the proposed design rules [27]. However, the CAS has been reduced when the ratio approached 1, validating the significance of the proposed methodology. Furthermore, the experimental results of the fabricated design are compared with the reported techniques as listed in Table 3, showing the contribution and significance of the proposed technique for CAS reduction.

## 6. Conclusions and Future Work

This work presents a designing technique for cross-axis sensitivity estimation and reduction in a single-drive multi-axis MEMS gyroscope using COMSOL Multiphysics. For a single-drive multi-axis MEMS gyroscope with a complex mechanical structure, it was not easy to estimate and reduce the cross-axis sensitivity using MATLAB or analytical approaches. However, COMSOL Multiphysics provides a more straightforward and easy approach to estimate and reduce cross-axis sensitivity of single-drive multi-axis MEMS gyroscope. Initially, x and y-axes cross-axis sensitivity was modeled and computed to be 0.482% and 0.120%, respectively, having an average of 0.301% in the designed gyroscope. The effect of coupling spring position on the CAS was analyzed by varying its position. Average cross-axis sensitivity of 0.091% was achieved at the center position for the coupling spring placement, showing that the CAS of the designed gyroscope depended on the coupling spring position. The center position of the x and y-coupled masses was considered as an optimum position for coupling spring placement.

A design technique, the “ratio-matching method”, based on the drive displacement amplitudes and sense frequency differences, was proposed to further reduce the cross-axis sensitivity in a single-drive multi-axis MEMS gyroscope. In the proposed “ratio-matching method”, drive displacement amplitudes were limited according to sense frequency differences by utilizing a Z-shaped coupling spring. Cross-axis sensitivity analysis was performed, and the average CAS was reduced to 0.045%, showing a reduction rate of 85.1% through the “ratio-matching method”. Individually, x and y-axes CAS was computed to be 0.018% and 0.073%, respectively. The proposed “ratio-matching method” was successfully validated by performing CAS analysis for coupling spring position variation, and it was noted that cross-axis sensitivity was reduced regardless of the coupling spring position whenever the ratio was approaching 1. However, minimum CAS was measured by placing a coupling spring at the center position of the sense masses.

The computed results and validation of the proposed ratio-matching method proved that the cross-axis sensitivity of a single-drive multi-axis MEMS gyroscope was dependent on the sense frequency difference, as well as the drive displacement amplitude. However, it is concluded from all the analyses that, for a single-drive multi-axis MEMS gyroscope with low cross-axis sensitivity, it was necessary to design the drive-scheme according to the sense frequency differences and place the coupling spring at the center position of the sense masses. Furthermore, the proposed “ratio-matching method” was experimentally validated using previously fabricated design of single-drive multi-axis MEMS gyroscope. Moreover, the previously developed FEA model was validated for cross-axis sensitivity evaluation of the single-drive multi-axis MEMS gyroscope. Nevertheless, the proposed design technique helps researchers in the design optimization and performance improvement of single-drive multi-axis MEMS gyroscope. The proposed methodology of CAS reduction will be implemented in MEMS vibratory gyroscope designs having equal drive displacement scheme in future work.

## Figures and Tables

**Table 1:** Resonant Frequencies of the Designed MEMS Gyroscope.

Frequency Mode		Frequency (Hz)	Frequency Difference (Hz)
$f_{dr}$	Drive mode	25,682	−−
$f_{cx}$	x-sense	25,979	$f_{dr}-f_{cx}=-297$
$f_{cy}$	y-sense	26,036	$f_{dr}-f_{cy}=-354$

**Table 2:** Cross-axis Sensitivity Analysis Results of the Designed MEMS Gyroscope.

Axis	Parameters		Value	Remarks
x-axis	$y_{s_{XX}}$(nm)	$M_{1}$	30.774	x-sense displacement of M_1_
		$M_{3}$	−30.799	x-sense displacement of M_3_
	Differential (nm)	$M_{3}-M_{1}$	61.573	x-sense total differential displacement
	$y_{s_{XY}}$(nm)	$P_{1}+P_{2}$	0.171	Two-point method for cross-axis displacement calculation
		$P_{3}+P_{4}$	−0.126	
	Differential (nm)	$S_{XY}$	0.297	Total differential cross-axis displacement in x-axis due to the y-axis
	$S_{X_{Cross}}$		0.482%	Cross-axis sensitivity in the x-axis
y-axis	$y_{s_{YY}}$(nm)	$M_{2}$	29.792	y-sense displacement of M_2_
		$M_{4}$	−29.640	y-sense displacement of M_4_
	Differential (nm)	$M_{4}-M_{2}$	59.432	y-sense total differential displacement
	$y_{s_{YX}}$(nm)	$P_{1}+P_{2}$	−0.036	Two-point method for cross-axis displacement calculation
		$P_{3}+P_{4}$	0.035	
	Differential (nm)	$S_{YX}$	0.071	Total differential cross-axis displacement in y-axis due to the x-axis
	$S_{Y_{Cross}}$		0.120%	Cross-axis sensitivity in the y-axis
Average cross-axis sensitivity			0.301%	Average CAS in Proposed design

**Table 3:** Experimental Results Comparison of Fabricated Design with Reported Gyroscope.

Methodology	Reference	Cross-Axis Sensitivity (%)		Improvement (%)	
		x-Axis	y-Axis	x-Axis	y-Axis
Ratio-Matching method	This Work	18.882	11.207	35.100	10.509
Integrated automatic gain control for drive mode	[20]	22.000	9.000	No details	
Quadrature error cancellation	[19]	25.200	20.100	No details	
